# Host-Targeted Therapeutics against Multidrug Resistant Intracellular *Staphylococcus aureus*

**DOI:** 10.3390/antibiotics8040241

**Published:** 2019-11-28

**Authors:** Natalia Bravo-Santano, Volker Behrends, Michal Letek

**Affiliations:** 1 Health Sciences Research Centre, University of Roehampton, London SW15 4JD, UK; nbravosantano@gmail.com (N.B.-S.); Volker.Behrends@roehampton.ac.uk (V.B.); 2 Department of Molecular Biology, Area of Microbiology, University of León, 24004 León, Spain

**Keywords:** *Staphylococcus aureus*, host-targeted, repurposing drugs, intracellular pathogen

## Abstract

*Staphylococcus aureus* is a facultative intracellular pathogen that invades and replicates within many types of human cells. *S. aureus* has shown to rapidly overcome traditional antibiotherapy by developing multidrug resistance. Furthermore, intracellular *S. aureus* is protected from the last-resort antibiotics—vancomycin, daptomycin, and linezolid—as they are unable to achieve plasma concentrations sufficient for intracellular killing. Therefore, there is an urgent need to develop novel anti-infective therapies against *S. aureus* infections. Here, we review the current state of the field and highlight the exploitation of host-directed approaches as a promising strategy going forward.

## 1. Methicillin-Resistant *Staphylococcus aureus* (MRSA) Methicillin-Resistant Staphylococcus Aureus as an Example of Antibiotic Resistance

Since antibiotics were discovered and introduced into the market, they have become a standard treatment for bacterial infections. Indeed, antibiotics are often administrated as a routine prophylactic measure during medical procedures where bacterial infection may occur (e.g., surgery) [1]. In addition, antibiotics are commonly used in agriculture to prevent bacterial infections and, also, as growth promoters [2]. This increasing exposure to antibiotics has contributed to the emergence of multidrug-resistant bacteria.

In particular, the ability to rapidly acquire antibiotic resistance represents one of the major features of *Staphylococcus aureus*. In 1959, methicillin was introduced to treat *S. aureus* infections caused by penicillin-resistant isolates. Two years later, in 1961, the first methicillin-resistant *S. aureus* isolate was reported in the United Kingdom [3]. Since then, several MRSA clones have been identified over the past decades [4]. In fact, many staphylococcal infections are caused by strains that are resistant to multiple antibiotics, which are associated with higher costs and extended hospitalization periods, as well as higher morbidity and mortality rates [5].

Moreover, although MRSA infections were initially identified as nosocomial infections, the number of MRSA infection cases within healthy community settings has risen in the past two decades, especially in the United States [6,7,8]. Community-acquired MRSA (CA-MRSA) and hospital-acquired MRSA (HA-MRSA) strains differ in their genotypic and phenotypic characteristics. For instance, CA-MRSA isolates are susceptible to most antimicrobials apart from β-lactam antibiotics and erythromycin, whereas HA-MRSA isolates are resistant to most available antibiotics [9].

## 2. Intracellular MRSA Is Protected from Common Antibiotic Treatments

The opportunistic and facultative intracellular pathogen *S. aureus* is carried by 30% of the global population [10,11], the anterior nares of the nasal cavity being the most common carriage site [12,13]. During nasal colonization, *S. aureus* is capable of internalizing into human nasal epithelial cells, and the colonization of the anterior nares increases the risk of developing bacteraemia in persistent carriers. Skin and soft tissue infections are another common portal of entry, which may lead to the colonization of the bloodstream, and, consequently, organ dysfunction and sepsis [14]. Intracellular *S. aureus* is also associated with recurrent rhinosinusitis, tonsillitis, and chronic osteomyelitis [11]

Host cell invasion and intracellular survival could be used by *S. aureus* to infect macrophages, spread to secondary points of infection, evade immune recognition, and avoid exposure to last-resort antibiotics [15,16]. Importantly, the serum levels that can be reached without causing toxicity of three last resort antibiotics routinely employed to treat MRSA infection—vancomycin, daptomycin, and linezolid—are not sufficient to achieve intracellular killing and the eradication of this pathogen [15]. As a result, patients are often required to receive long treatments of intravenous vancomycin, which is in stark contrast to the in vitro effective killing of *S. aureus* observed for this antibiotic [17]. Moreover, clinical infection relapse is not uncommon, suggesting that the intracellular survival of these bacteria facilitates their resistance to the immune system and current antibiotherapies [18]. In fact, antibiotic treatment failure occurs in 20% of patients, leading to an estimated 20,000 deaths per year in the United States alone [19], despite the fact that the clinical isolates often show sensitivity to the administered antibiotics [20]. 

## 3. Current Clinical Management of *S. aureus* Infections

Treatment of *S. aureus* infections is becoming a real challenge, especially considering the emergence of MRSA strains resistant to last-resort antibiotics (i.e., vancomycin) [21], as well as its protection against current antibiotics once internalized [15]. 

Clinical management of MRSA infections varies depending on the type of infection, as well as the bacterial strain. Overall, most MRSA infections usually require a prolonged period of antibiotic therapy, and the removal of infected tissue or biomaterial in cases of localized infection or prosthetic joint infections, respectively [22]. 

The current list of antibiotics available and approved to treat MRSA infections are vancomycin, daptomycin, linezolid, and some other antimicrobials that have been recently developed, including tedizolid, telavancin, oritavancin, dalbavancin, ceftaroline, and ceftobiprole [23]. However, these latter antibiotics are mostly employed to treat skin and soft tissue infections, with vancomycin, daptomycin, and linezolid being the top options for non-topical and/or systemic infections. Nevertheless, these three last-resort antibiotics also present some limitations. For example, daptomycin cannot be used to treat pneumonia [24], whereas linezolid is not suitable for bacteraemia or infective endocarditis treatment [25]. In addition, strains with reduced susceptibility to daptomycin and linezolid, as well as vancomycin-intermediate and vancomycin-resistant *S. aureus* (VISA and VRSA) strains have already been reported [26]. In addition, and as discussed above, effective doses of vancomycin, daptomycin, or linezolid cannot be achieved intracellularly without causing toxicity to the patient [15].

Furthermore, it is becoming clear that the complex interaction between the pathogen and the host immune system is preventing the development of an efficient vaccine. For example, different staphylococcal virulence factors, such as protein A, possess immunosuppressive effects, which compromise the immunological memory of the host [27]. Indeed, it has been recently demonstrated that vaccination against staphylococcal antigens located on the cell surface may even aggravate the infection [28]. Another important issue regarding the development of a *S. aureus* vaccine is the use of experimental animals that are not natural hosts of this pathogen [29]. For instance, although some protection against *S. aureus* can be obtained in mice, the translation of this protective effect into humans has failed repeatedly [27].

Therefore, due to the alarming emergence of antibiotic resistance, the lack of a suitable vaccine against *S. aureus* infections and the intracellular protection from last-resort antibiotics, novel therapeutics capable of targeting intracellular *S. aureus* are urgently needed. A recently explored strategy to tackle this problem is the design of antibodies conjugated with bactericidal antibiotics that are specifically released within the host cell [15]. However, in this approach, the antibiotics are only active due to the lower pH of the phagosome, suggesting that this antibody–antibiotic complex may not be effective against cytosolic *S. aureus* or replicating bacteria within autophagosomes. In addition, the use of antibiotics targeting cell growth or division of the pathogen is still a major selective pressure towards the emergence of resistant strains.

## 4. Host-Directed Therapies: A Novel Perspective

A recent study has estimated that the development of a new drug that gets market approval costs almost 3 billion United States dollars [30], and this is a process that takes 10–12 years on average [31]. Taking into account the limited lifetime of antibiotics, the consideration of alternative therapeutic strategies aimed at improving the activity of currently available antibiotics might be the only realistic option to combat the antibiotic crisis in the short, medium, and long term [32,33].

As part of their host-defense evasion mechanism, intracellular pathogens—including *S. aureus*—subvert and exploit a wide range of host factors to support their intracellular survival, targeting multiple pathways to assure their intracellular proliferation [17,34]. Hence, the study of host–pathogen interactions may lead to the identification of potentially novel pathogen-specific drug targets and/or host-directed therapeutics [17,35].

Repurposing commercially available drugs that target host pathways hijacked by intracellular pathogens is a particularly interesting strategy [33]. The main advantage of using repurposed drugs is that they show minimal toxicity to the host cell, and that they have already been approved for other clinical purposes, which would significantly reduce the necessary time to have these drugs in the market [34]. 

In addition, the use of repurposed host-targeted therapeutics in combination with traditional antibiotherapy may increase the lifetime of currently available antibiotics by preventing intracellular pathogens’ escape from antibacterial treatment within host cells. This may shorten the length of the treatment required for chronic infections and it should lower the risk of infection relapse.

Indeed, the research focused on host-directed therapies has already identified many drugs that have shown positive effects against intracellular bacterial pathogens (Table 1). These examples provide proof-of-principle about the potential of this approach to improve the outcome of bacterial infections caused by these pathogens. Such therapies have shown the ability to achieve intracellular killing, and they also possess the benefit of preventing the development of antimicrobial resistance [36,37]. 

Host-directed approaches require a deep understanding of the host–pathogen interactions. In order to gain novel insights about such interactions, individual or combined strategies that include-omics approaches (genomics, transcriptomics, proteomics, and metabolomics) and computational biology have shown great promise. Certainly, genome-wide screens employing RNA interference (RNAi), microarray analyses, and chemical libraries have already been employed to identify host genes required for bacterial entry and intracellular growth of intracellular pathogens such as MRSA [35].

RNAi approaches allow loss-of-function phenotypic analyses to discover novel host-directed targets. For instance, genome-wide RNA interference assays have been performed in *Drosophila* cells to identify host factors exploited and subverted by two intracellular pathogens: *Chlamydia caviae* and *Listeria monocytogenes*. In *C. caviae* infections, the depletion of human genes *TOMM40* or *TOMM22*, which are involved in protein transport to the mitochondria, resulted in a reduction of intracellular bacteria within mammalian cells [79]. Moreover, 116 host genes were identified to be important during *L. monocytogenes* infection and implicated in entry, phagosomal escape, and intracellular replication [80].

Interestingly, a study that employed a genetic approach combining RNAi and automated microscopy revealed the importance of serine-threonine protein kinases (Akt1) in the intracellular survival of several intracellular pathogens. Akt1 kinase acts as a key regulator of several guanosine triphosphatases (GTPases), which are involved in essential host pathways exploited by intracellular pathogens. For example, *Salmonella* Typhimurium is able to activate host Akt1 to control actin dynamics via p21(Cdkn1a)-activated kinase 4 (PAK4) and, hence, facilitate its intracellular survival [50]. Moreover, we recently screened for novel host targets that are essential for the host cell infection of MRSA by means of functional genomics. More specifically, we employed an unbiased short-hairpin RNA (shRNA) lentiviral library to screen 16,000 human genes during intracellular *S. aureus* infection, identifying several host genes important for intracellular MRSA [81]. In particular, we found that silencing the human gene *TRAM2* resulted in a significant reduction of intracellular MRSA, whereas host cell viability was restored, showing its importance during intracellular infection. TRAM2 is an interactive partner of the sarco/endoplasmic reticulum Ca^2+^ ATPase (SERCA) [82]. Accordingly, we found that very low doses of thapsigargin (an inhibitor of the SERCA pumps) could be used to stop *S. aureus* intracellular survival in combination with conventional antibiotics [81].

The use of metabolomics also represents an interesting approach to gain a better understanding of the metabolic scenario upon intracellular bacterial infection [83,84]. It has been shown that several intracellular pathogens trigger host cell metabolism changes to support its intracellular survival [85]. For example, *Legionella pneumophila* induce a Warburg-like effect in the host cell by interacting with mitochondria to favour its own replication [86]. *Shigella flexneri* re-routes host central carbon metabolism to obtain an abundant nutrient-flux through the glycolytic pathway that allows its intracellular survival [87]. Moreover, host cholesterol import is required by intracellular *Mycobacterium tuberculosis* to persist inside both macrophages and mice lungs [88]. On the other hand, a reduction in nutrient uptake as well as nucleotide biosynthesis has been recently observed in human airway epithelial cells infected with *S. aureus* [89].

We recently characterized host cell physiology of MRSA-infected cells using mass spectrometry-based metabolomics [17,90]. We found that *S. aureus* infection leads to starvation-induced autophagy due to a block in central carbon metabolism, which is mediated by the energy sensor AMPK (AMP-activated protein kinase). Consequently, a treatment with the AMPK inhibitor dorsomorphin halted intracellular *S. aureus* in HeLa and Human Umbilical Vein Endothelial Cells (HUVECs) [17]. Accordingly, treatments with autophagy inhibitors protect mice from MRSA pneumonia [91].

Once specific host factors or metabolic pathways required by the intracellular pathogen have been described, blocking these pathways—for example, by using drug inhibitors—may be considered as a novel strategy to control bacterial infections. For example, Akt1 and protein kinase A (PKA) inhibitors have been shown to reduce intracellular load of *M. tuberculosis* and *S. aureus* Typhimurium in infected human macrophages [50]. Moreover, host-directed therapies could also aim to enhance host cellular responses against intracellular pathogens to activate innate and adaptive host immune responses or to modulate disproportionate inflammation. Hence, host-directed therapies also include immunomodulatory agents—including monoclonal antibodies or nutritional products—as well as cellular therapy (Table 1). For example, monoclonal antibodies anti-TNFα and anti-interleukins, which reduce tissue-destructive inflammation by cytokine neutralisation, have shown a bactericidal effect against the intracellular pathogens *M. tuberculosis* and *Helicobacter pylori* [38,39,92]. Further, the nutritional product vitamin D3 is also effective against these two pathogens, and its mechanism of action has been described as an activation and enhancement of host antimicrobial defences [78,93].

In addition, several studies have already shown that existing and approved drugs, which are used for other clinical purposes unrelated to infection biology, can influence the outcome of bacterial infections. The main advantage of finding “repurposed drugs” is that they are already clinically approved, shortening the time to reach the clinic [35,53]. An example of repurposed drug is the histone deacetylase inhibitor sulforaphane, which induces the expression of leukocyte protease inhibitor and β-defensin 2 and, therefore, increases the activity of antibiotics against the multidrug-resistant strain *Neisseria gonorrhoeae* [70]. Similarly, imatinib—an ABL tyrosine kinase inhibitor, commercialized as Gleevec—is under early clinical trials to treat complicated infections caused by *M. tuberculosis* [57,94].

To further identify other host-targeted therapeutics against intracellular MRSA, we recently screened a panel of 133 FDA-approved drugs with known host targets to test whether blocking these targets had an effect on intracellular bacterial survival [34]. Interestingly, we found that ibrutinib, a host kinase inhibitor, significantly increased host cell viability and reduced bacterial survival at clinically relevant concentrations. We also determined the mechanism of action of ibrutinib by mass spectrometry-based phosphoproteomics, finding very promising host targets, such as Ephrin receptor 2 (EPHA2), that could be used to further develop a more specific therapy against intracellular *S. aureus* [34]. 

In summary, Table 2 compresses the host factors that have been identified as important for intracellular *S. aureus* internalization, survival, and/or replication. It also includes the putative function of each pathway and the potential host-directed drug (if known) to tackle such pathway with the aim to impair intracellular *S. aureus*.

## 5. Conclusions

Future anti-infective therapies may consist in the combination of conventional antibiotics targeting bacterial survival outside of the host cell with host-directed therapies to efficiently eliminate intracellular pathogens. Therefore, a better understanding of host–*S. aureus* interactions during intracellular infection may lead to novel therapeutic strategies by targeting those host cellular pathways exploited by this versatile pathogen.

## Figures and Tables

**Table 1:** List of host-directed drugs able to halt the intracellular infection caused by different pathogens.

Class	Drug	Mechanism of Action	Pathogen	Reference
Monoclonal antibody	Adalimumab	Anti-TNFα	*Mycobacterium tuberculosis*	[38]
	Anti-interleukin 1β	Cytokine neutralisation	*Helicobacter pylori*	[39]
	Antipertussis toxins antibody	Enhancement of immunoglobulins	*Bordetella pertussis*	[40]
	Anti-TNFα	Cytokine neutralisation	*Helicobacter pylori*	[39]
	Bevacizumab	Anti-VEGF	*Mycobacterium tuberculosis*	[41,42]
	Nivolumab	Anti-PD-1	*Mycobacterium tuberculosis*	[43,44]
	Siltuximab	Anti-interleukin 6	*Mycobacterium tuberculosis*	[45]
Repurposed drug	Aspirin	NSAID, TNFα levels reduction	*Mycobacterium tuberculosis*	[46]
	Chlorpromazine	Calmodulin antagonist	*Staphylococcus aureus*	[47]
			*Neorickettsia risticii*	[48]
			*Salmonella* Typhimurium	[49]
	ETB067	Serine-threonine protein kinase (Akt1) inhibitor	*Mycobacterium tuberculosis*	[50]
	Fingolimod	Activation of sphingosine-1-phosphate pathway	*Bordetella pertussis*	[51]
	Glibendamide	Cyclooxygenase inhibition	*Streptococcus pneumoniae*	[52]
	H-89	Protein kinase A (PKA) inhibitor	*Salmonella* Typhimurium	[50]
			*Coxiella burnetii*	[53]
	Ibuprofen	NSAID, cyclooxygenase inhibition	*Streptococcus pneumoniae*	[54]
			*Mycobacterium tuberculosis*	[55,56]
	Imatinib mesylate	BCR-ABL tyrosine kinase inhibitor	*Mycobacterium tuberculosis*	[57]
			*Anaplasma phagocytophilum*	[58]
Repurposed drug	Indometacin	Cyclooxygenase inhibition	*Streptococcus pneumoniae*	[59]
	Metformin	Mitochondrial respiratory chain blocker	*Mycobacterium tuberculosis*	[60]
	Niraparib	PARP inhibitor	*Mycobacterium tuberculosis*	[61]
	Phenylbutyrate	Histone deacetylase inhibitor	*Mycobacterium tuberculosis*	[62]
	Pimozide	Calcium channel inhibitor	*Listeria monocytogenes*	[63]
			*Bacillus subtilis*	[63]
			*Salmonella* Typhimurium	[63]
			*Escherichia coli*	[63]
	Prednisone	Glucocorticoid receptor antagonist	*Streptococcus pneumoniae*	[64]
			*Mycobacterium tuberculosis*	[65]
	Raloxifene	Oestrogen receptor modulator	*Pseudomonas aeruginosa*	[66]
	Statins	HMG-CoA reductase inhibitor	*Streptococcus pneumoniae*	[67,68]
			*Mycobacterium tuberculosis*	[69]
	Sulforaphane	Histone deacetylase inhibitor	*Neisseria gonorrhoeae*	[70]
	Thapsigargin	Calcium ATPase inhibitor	*Coxiella burnetii*	[53]
	Thioridazine	unknown	*Listeria monocytogenes*	[71]
			*Staphylococcus aureus*	[47]
			*Mycobacterium tuberculosis*	[72]
	Verapamil	Calcium channel inhibitor	*Mycobacterium tuberculosis*	[73,74]
			*Chlamydia pneumoniae*	[75]
	Vorinostat	Histone deacetylase inhibitor	*Mycobacterium tuberculosis*	[76]
	Zileuton	Leukotriene synthesis inhibitor	*Mycobacterium tuberculosis*	[77]
Vitamin	Vitamin D3	Activation of antimicrobial defenses	*Helicobacter pylori*	[78]

**Table 2:** Host molecular factors hijacked by *S. aureus* during intracellular infection and potential host-directed drugs against intracellular *S. aureus*.

Host Factor	Putative Function	Reference	Host-Directed Drug	Type
**Adherence and Internalization**				
α5β1-integrins	Internalization into non-phagocytic cells	[95]	Volociximab *	Antibody
FAK	Internalization into non-phagocytic cells	[96,97]	PF-562271	Inhibitor
Src-mediated cortactin	Internalization into non-phagocytic cells	[96,97]	PP2	Inhibitor
β_1_-integrins	Internalization into mast cells	[98]	R1295 *	Antagonist
Annexin 2	Internalization into epithelial cells	[99]		
(PI3K)-Akt	Internalization into endothelial cells	[100]	Nelfinavir *	Inhibitor
αVβ3-integrin	Internalization into endothelial cells	[101]	Cilengitide *	Inhibitor
ERK	Internalization into osteoblast and Hep-2 cells	[102]	SCH772984 *	Inhibitor
ERK1/2/MEK	Penetration into airway epithelial cells	[103]	UO126	Inhibitor
ERK	Invasion to fibroblasts	[104]	PD98059	Inhibitor
Hsp60	Internalization into epithelial cells	[105]		
Hsc70	Internalization into 293T cells	[106]		
Desmoglein 1	Adherence to keratinocytes	[107]		
Scavenger protein gp340	Internalization into A549 cells	[108]		
EGFR	Penetration into airway epithelial cells	[103]	BPDQ	Inhibitor
ROCK	Penetration into airway epithelial cells	[103]	Y-27632	Inhibitor
JNK	Penetration into airway epithelial cells	[103]	SP600125	Inhibitor
p38/MAPK	Penetration into airway epithelial cells	[103]	SB202190	Inhibitor
EPHA2	Invasion/Internalization into epithelial cells	[34]	Ibrutinib	Inhibitor
CDK	Adhesion to human bronchial epithelial cells	[97]	Roscovitine	
PKA	Internalization into Thp1 macrophages	[97,109]	H-89	Inhibitor
PKC	Internalization into Thp1 macrophages	[97,109]	Bisindolylmaleimide-I	Inhibitor
**Intracellular Survival and Proliferation**				
ADAM10	Cleavage of adherens junction protein E-cadherin	[110]	GI 254023X	Inhibitor
AMPK	Induction of autophagy	[17]	Dorsomorphin	Inhibitor
ERK	Induction of autophagy	[17]	SCH772984 *	Inhibitor
TRAM2	Ca2+ pump to promote collagen synthesis	[81]	Thapsigargin	Inhibitor
p38/MAPK	Subversion of autophagy	[111]	Skepinone-L *	Inhibitor
PAK	Cytoeskeleton rearrangements	[97]	FRAX597 *	Inhibitor
MYL2	Cytoeskeleton rearrangements	[81]	Blebbistatin *	Inhibitor
FAM63B	Intracellular trafficking	[81]		
Actin	Promote bacterial movements within the host cell	[95,112]	Cytochalasin D	Inhibitor
Rab5	Promote bacterial movements within the host cell	[112]		
NWASP	Production of actin-comet tails to facilitate movement	[112]	Wiskostatin	Inhibitor
TMEM59	Activation of selective-autophagy	[113]		
RAPGEF3	Induction of autophagy	[114]	Salirasib *	Inhibitor
RAP2B	Induction of autophagy	[114]		
***S. aureus*-Induced Host Cell Death**				
Caspase 2	*S. aureus*-induced apoptosis	[115]	Z-VDVAD-FMK	Inhibitor
Caspase 9	*S. aureus*-induced apoptosis	[116]	Z-LEHD-FMK	Inhibitor
NLRP3	*S. aureus*-induced pyronecrosis	[117]	MCC950 *	Inhibitor

*: Drugs that could potentially halt intracellular *S. aureus* by targeting the described host pathway, but their effects have not been yet investigated.
